# Residual γH2AX foci as an indication of lethal DNA lesions

**DOI:** 10.1186/1471-2407-10-4

**Published:** 2010-01-05

**Authors:** Judit P Banáth, Dmitry Klokov, Susan H MacPhail, C Adriana Banuelos, Peggy L Olive

**Affiliations:** 1 Medical Biophysics Department, BC Cancer Agency Research Centre, 675 W. 10th Ave., Vancouver, B.C., V5Z 1L3, Canada; 2 Radiological Protection Research and Instrumentation, Atomic Energy of Canada Ltd, CHL Stn 51, Chalk River, Ont., Canada

## Abstract

**Background:**

Evidence suggests that tumor cells exposed to some DNA damaging agents are more likely to die if they retain microscopically visible γH2AX foci that are known to mark sites of double-strand breaks. This appears to be true even after exposure to the alkylating agent MNNG that does not cause direct double-strand breaks but does produce γH2AX foci when damaged DNA undergoes replication.

**Methods:**

To examine this predictive ability further, SiHa human cervical carcinoma cells were exposed to 8 DNA damaging drugs (camptothecin, cisplatin, doxorubicin, etoposide, hydrogen peroxide, MNNG, temozolomide, and tirapazamine) and the fraction of cells that retained γH2AX foci 24 hours after a 30 or 60 min treatment was compared with the fraction of cells that lost clonogenicity. To determine if cells with residual repair foci are the cells that die, SiHa cervical cancer cells were stably transfected with a RAD51-GFP construct and live cell analysis was used to follow the fate of irradiated cells with RAD51-GFP foci.

**Results:**

For all drugs regardless of their mechanism of interaction with DNA, close to a 1:1 correlation was observed between clonogenic surviving fraction and the fraction of cells that retained γH2AX foci 24 hours after treatment. Initial studies established that the fraction of cells that retained RAD51 foci after irradiation was similar to the fraction of cells that retained γH2AX foci and subsequently lost clonogenicity. Tracking individual irradiated live cells confirmed that SiHa cells with RAD51-GFP foci 24 hours after irradiation were more likely to die.

**Conclusion:**

Retention of DNA damage-induced γH2AX foci appears to be indicative of lethal DNA damage so that it may be possible to predict tumor cell killing by a wide variety of DNA damaging agents simply by scoring the fraction of cells that retain γH2AX foci.

## Background

Several DNA repair pathways have evolved to maintain cell viability after exposure of mammalian cells to DNA damaging agents. Sufficiently high doses of drugs or radiation cause cell killing, and it seems reasonable to expect that those cells that can repair DNA damage will survive while those unable to repair their damage will die. Sensitive detection of residual DNA damage at the level of the individual cell could allow us to identify treatment resistant subpopulations within tumors. This possibility can now be examined by making use of the fact that complex DNA lesions such as DNA double-strand breaks (DSBs) are marked by microscopically visible γH2AX foci [1].

DSBs rapidly activate kinases that phosphorylate histone H2AX. Resulting γH2AX foci can be used to identify the number and location of DSBs and to follow their fate during recovery [2,3]. The fraction of tumor cells that retain γH2AX foci 24 hours after irradiation has been correlated with the fraction of cells that fail to divide and form colonies [4,5]. Similar results have been reported for RAD51 recombinase, a key player in DSB repair by homologous recombination [6]. RAD51 molecules also accumulate slowly as microscopically visible foci that are often co-expressed in cells with γH2AX foci [7,8]. Recently, RAD51 foci have been found in association with persistent DSBs [9]. What is not known for certain is whether the cells that retain γH2AX or RAD51 foci 24 hours after irradiation are actually the cells that die.

γH2AX foci begin to form immediately after irradiation, reaching a maximum size about 30 or 60 min later and disappearing over the next several hours. However, residual foci may remain in some cells for days after exposure and may mark unrepaired or misrepaired sites [10,11]. Importantly, residual foci appear to be replicated and retained by daughter cells [4,5]. Since rapid loss of γH2AX is contingent upon functional DNA repair, it is not surprising that retention of γH2AX foci has been associated with loss of clonogenic potential. Several studies have reported that repair-deficient cell lines retain more foci and more cells with foci when analyzed 24 hours after irradiation [12,13]. The percentage of cells that retained γH2AX foci 24 hours after irradiation was correlated with the percentage of cells that lost clonogenicity, thus making it possible to use the fraction of cells with residual foci as a way to estimate sensitivity to killing by ionizing radiation [4,5].

DSBs may be produced either directly or indirectly [2]. Direct DSBs occur as a result of exposure to ionizing radiation as well as selected drugs including bleomycin and the topoisomerase II inhibitor, etoposide. Indirectly produced DSBs can arise when a single-strand break, crosslinked DNA, or damaged DNA base meets a replication fork [14]. Phosphorylation of H2AX may also occur indirectly during repair of base damage [15]. Arguably also an indirect mechanism, extensive H2AX phosphorylation occurs as a result of DNA fragmentation during the process of apoptosis [16]. Therefore directly or indirectly, the majority of DNA damaging agents are likely to cause H2AX phosphorylation, and cells that subsequently retain γH2AX foci may be more likely to die no matter how the DNA damage was initially produced. To explore this possibility, the fraction of cells that retained γH2AX foci was compared to the fraction of clonogenic surviving cells measured after a short exposure to 8 drugs known to damage DNA and cause H2AX phosphorylation [2].

A correlation between clonogenicity and fraction of cells lacking foci does not constitute proof that cells that retain γH2AX foci are the cells that will die. Real-time imaging of γH2AX foci is complicated by the necessity of identifying the phosphorylated form of H2AX. However, RAD51 molecules also aggregate as clusters at sites of DNA damage in irradiated cells and are retained by γH2AX [10]. When labeled with green fluorescent protein (GFP), RAD51-GFP can be used for live cell analysis to determine the fate of an individual cell that retains RAD51 foci [17,18]. The ability to follow live cells allowed a direct test of the hypothesis that cells that retain RAD51 foci 24 hours after irradiation are the cells that will eventually die.

## Methods

### Cell lines and drug treatment

Chinese hamster V79 and CHO cells were maintained by twice weekly sub-cultivation in minimal essential medium (MEM) containing 10% fetal bovine serum (FBS). SiHa human cervical carcinoma cells and HT144 human melanoma cells were obtained from American Type Culture Collection. SKOV3 human ovarian carcinoma cells were obtained from the DCTD tumor repository in Frederick MD. M059J and M059K human glioma cell lines were obtained from Dr. J. Allalunis-Turner, Cross Cancer Center. All tumor cell lines were sub-cultured twice weekly in MEM containing 10% FBS.

To obtain cells that expressed RAD51-GFP, SiHa cells were transfected with a plasmid kindly supplied by Dr. Roland Kanaar [18]. Transfection was accomplished using Lipofectamine Plus using the protocol supplied by Invitrogen; stably transfected cells were selected by growth in 200 μg/ml G418 (Gibco), and a clone was chosen for further studies.

For drug treatment, 5 × 10^5 ^cells/60 mm dish and were exposed as exponentially growing monolayers to selected drugs usually for 30 min or for 60 min (cisplatin, temozolomide) in medium containing 5% FBS. Tirapazamine treatment was conducted using cells in suspension culture incubated for 30 min in drug-containing medium pre-equilibrated for one hour with 95% oxygen and 5% CO_2_. Cisplatin was obtained from Mayne Pharma and diluted from a stock solution of 1 mg/ml. Temozolomide was prepared in DMSO using a 250 mg capsule from Schering Canada. Etoposide was purchased from Novopharm and diluted from a stock concentration of 20 mg/ml. Doxorubicin, MNNG and hydrogen peroxide (H2O2) were obtained from Sigma and diluted in medium. Camptothecin was purchased from GBiosciences and prepared from a stock solution of 2 mM in DMSO. Tirapazamine was supplied by Dr. J. Martin Brown, and diluted from a stock solution of 2.5 mM in phosphate buffered saline. For experiments using X-rays, cells were exposed using a 300 kV unit at a dose rate of 5.2 Gy/min.

After drug incubation, drug was removed, dishes were rinsed several times, and cells were incubated for 24 hours in fresh complete medium. Trypsin treatment (0.1% for 5 min) was used to produce a single cell suspension. Samples of single cells were plated in duplicate to measure colony formation and resulting colonies were stained and counted two weeks later. The survival of the treated cells was normalized to the plating efficiency of the non-treated cells. Experiments were performed 2-4 times using a range of drug doses. The remaining cells were fixed in 70% ethanol for analysis by flow or image cytometry to measure γH2AX and RAD51 antibody binding.

### Flow Cytometry for γH2AX

Antibody staining was performed as previously described using mouse monoclonal anti-phosphoserine-139 H2AX antibody (Abcam #18311; 1:4000 dilution) [19]. After secondary antibody labelling with Alexa-488 conjugated goat anti-mouse IgG, cells were rinsed and resuspended in 1 μg/mL 4',6-diamidino-2-phenylindole dihydrochloride hydrate (DAPI; Sigma), a UV-excitable DNA stain. Samples were analyzed using a dual-laser Coulter Elite flow cytometer using UV and 488 nm laser excitation. The γH2AX signal was divided by DNA content per cell to account for differences in cell cycle distribution, and results were normalized relative to untreated controls within each experiment. Normalized γH2AX intensity is reported for all of the cells within the population.

### Live cell analysis of RAD51-GFP

On average, one SiHa-RAD51-GFP cell was seeded into each well of an 8 well chamber slide with coverslip bottoms (Nalge Nunc International) containing 100 μl fresh complete medium and 100 μl conditioned medium prepared by filtering the complete medium recovered after two days of growth with high density cell cultures. Cells were allowed 4 hours to attach before exposing the chamber slide to 0 or 3 Gy and returning the chambers to the incubator for 24 hours. After 24 hours, each well was examined using a Zeiss inverted microscope with a 63× objective. To ensure high plating efficiency, analysis was restricted to cell doublets since this indicated that cells had attached and divided in the 24 hours period after irradiation. Images of cell doublets were obtained under phase and 488 nm excitation, and the location of the doublet in the well was noted. Almost invariably, both cells of a doublet exhibited RAD51-GFP foci or foci were absent from both cells at 24 hours. After scoring for the presence of foci, chamber slides were returned to the incubator for 2 weeks to allow time for colonies with greater than 50 cells to form. Approximately 40 doublets with foci and 40 doublets without foci were scored for clonogenicity.

### Immunohistochemistry for RAD51 and γH2AX

Antibody stained cells prepared for flow cytometry as described above were cytospun onto microscope slides. Alternatively, cells grown on coverslips were fixed for 20 min in 2% freshly prepared paraformaldehyde before incubating with mouse monoclonal antibodies against γH2AX (Upstate, 1:500 dilution) and/or rabbit polyclonal antibodies against RAD51 (Calbiochem, 1:500 dilution or Oncogen Sciences, 1:100 dilution). Cells were viewed using a Zeiss epifluorescence microscope using a 100× Neofluor objective and images were analyzed for foci/nucleus. Analysis of foci/nucleus was always concluded before analysis of clonogenicity so that objectivity of scoring using image analysis was maintained. Experiments were repeated 3 times and independent results for each sample (clonogenic fraction and # cells lacking foci) were plotted.

### Comet assays

Alkaline and neutral versions of the comet assay were used to measure MNNG-induced DNA single-strand breaks and DSBs respectively. Exponentially growing V79 hamster cells were exposed to MNNG for 30 min and then embedded in low gelling temperature agarose on a microscope slide. For the alkaline comet assay, slides were placed in a high-salt lysis solution for 1 h at pH 12.3 as previously described [20]. The neutral comet assay was performed using a 4 h lysis at 50°C, pH 8.3, as previously described [21]. For each drug dose and time, 150 comet images were analysed for DNA content, tail moment and percentage of DNA in the comet tail. Mean values are shown.

## Results

### Comparison between γH2AX formation, DNA break induction and cell killing by MNNG

**Figure 1:**
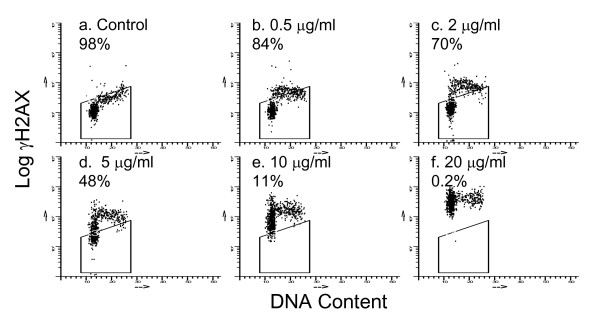
**Development of γH2AX after MNNG treatment**. Flow cytometric analysis was used to detect γH2AX formation in V79 cells exposed to MNNG for 30 min and then allowed 1 h to develop γH2AX foci. Single cells were fixed and analyzed for γH2AX antibody binding in relation to DNA content using flow cytometry. Cells that expressed control levels of γH2AX are contained within the boxes and are given as percentages.

Flow cytometry analysis of H2AX phosphorylation is a practical method to assess the kinetics of phorphorylation of this DNA damage reporter as well as the importance of cell cycle position. Flow cytometry profiles of γH2AX expression versus DNA content indicated that V79 cells respond differently to low and high doses of MNNG when examined 1 h after a 30 min exposure to the drug. Initial accumulation of γH2AX was limited to cells with S phase DNA content after treatment with low drug doses, consistent with replication fork collapse as a cause of H2AX phosphorylation (Fig. 1a-c). However, after exposure to higher drug doses, γH2AX developed in cells in all phases of the cell cycle (Fig. 1d-f).

**Figure 2:**
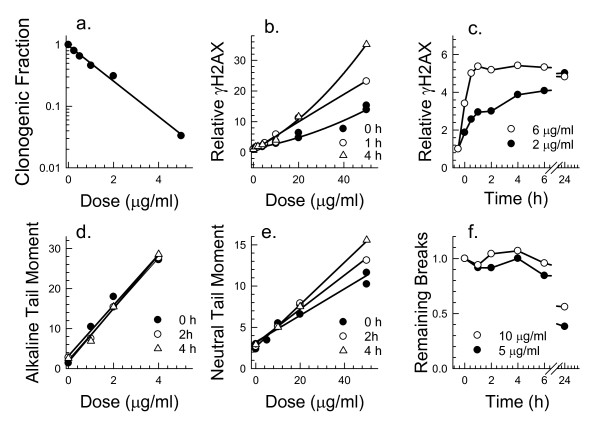
**Toxicity, DNA damage, and γH2AX formation in V79 cells exposed to MNNG**. Panel a: Clonogenic survival after a 30 min exposure of V79 cells to MNNG. Panel b: Expression of γH2AX in V79 cells relative to untreated cells as a function of dose of MNNG. Panel c: Time-dependence of γH2AX formation in V79 cells after exposure to low doses of MNNG. Panel d: Alkali-labile lesions measured using the alkaline comet assay showing drug dose dependence for different times of recovery after a 30 min exposure. Panel e: DNA double-strand breaks measured using the neutral comet assay as a function of drug dose and time after a 30 min exposure. Panel f: Rejoining of alkali-labile lesions as a function of time after exposure of V79 cells to MNNG.

Since our goal was to determine whether retention of γH2AX might be useful as an indicator of cell viability after exposure to genotoxic drugs, the loss of clonogenic ability was measured following a 30 min exposure to MNNG. Exponential cell killing was observed for V79 cells treated for 30 min with 0 to 5 μg/ml MNNG (Fig. 2a). A dose dependent increase in the population average expression of γH2AX was also detected using flow cytometry, and phosphorylation of H2AX continued to rise for several hours after exposure (Fig. 2b, c). This is consistent with γH2AX formation occurring as damaged DNA undergoes replication. By 24 hours after a 30 min drug treatment, the expression of γH2AX remained high (Fig. 2c) suggesting that many sites marked by γH2AX foci remained unrepaired. Nonetheless, when analyzed using flow cytometry, 7-22% of the cells within these populations expressed control levels of γH2AX (data not shown), consistent with the measured clonogenicity of these populations.

MNNG is an alkylating agent known to produce both DNA single-strand breaks as well as damage to the DNA bases. Production of alkali-labile DNA lesions including single-strand breaks and base damage was measured using the alkaline comet assay, a gel electrophoresis method that measures migration of broken DNA strands from individual nuclei. Tail moment, a measure of the amount and distance of DNA migration, was dose dependent, and there was no indication that the number of alkali-labile sites increased with time after exposure (Fig. 2d). Since γH2AX is formed in response to direct or indirectly-produced double-strand breaks and not single-strand breaks, the presence of double-strand breaks was also measured using a neutral version of the comet assay. Results indicated a small increase tail moment as a function of time after treatment with much higher doses (Fig. 2e), consistent with the observed increase in γH2AX (Fig. 2b). A comparison of the slopes in Fig. 2d and 2e indicated that approximately 40 times more alkali-labile lesions were produced than DNA double-strand breaks. Rejoining of alkali-labile lesions was negligible over the first few hours; however, half of the breaks were rejoined by 24 hours after treatment (Fig. 2f). The lack of a decrease in average γH2AX measured for all cells within the population after 24 hours could suggests that much of this repair, even after exposure to low doses, may not have been accurate or that a subset of lesions (e.g., single-strand breaks) that were repaired did not give rise to γH2AX foci. As residual γH2AX is a measure of the loss of foci as well as possible formation of new foci, the lack of a decrease in γH2AX does not necessarily indicate lack of repair of DNA breaks. Nonetheless, no rejoining of DNA double-strand breaks was detected after exposure to 50 μg/ml MNNG (data not shown). These results confirm that MNNG induces double-strand breaks that can be detected using the neutral comet assay but only after exposure to supra-lethal doses. The neutral comet assay lacks the sensitivity to predict response to MNNG in the low dose region.

**Figure 3:**
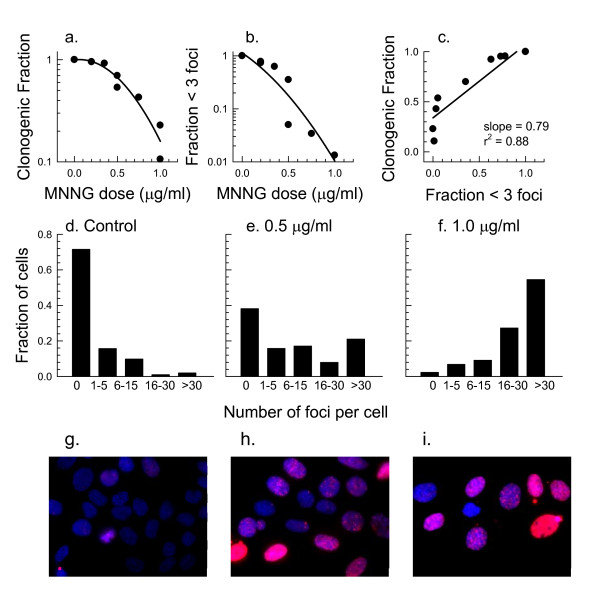
**Toxicity in relation to residual γH2AX in SiHa cells exposed to MNNG**. Panel a: Clonogenic surviving fraction after a 30 min exposure of SiHa cells to MNNG followed by a 24 hours recovery period. Panel b: Fraction of cells without γH2AX foci 24 hours after exposure to MNNG. Results are expressed relative to the endogenous expression of γH2AX; typically 60-70% of SiHa cells lack foci. Panel c: Comparison between fraction of cells lacking residual γH2AX foci and clonogenic fraction. Panels d-f: Distribution of γH2AX foci per cell. Panels g-i: Representative antibody-stained images of cells exposed 0, 0.5 and 1 μg/ml MNNG showing foci numbers and distribution.

The relation between clonogenic surviving fraction (Fig 3a) and fraction of cells with residual γH2AX foci (Fig. 3b) was then compared using SiHa human cervical carcinoma cells exposed for 30 min to MNNG. To improve resolution for detecting the relevant resistant cells within the population, the fraction of nuclei lacking foci was scored microscopically 24 hours after exposure to MNNG. The fraction of cells lacking foci at 24 hours was then compared with the fraction of cells from the same treated population that retained clonogenicity as measured two weeks later. A good correlation was observed between clonogenicity and fraction of cells lacking foci 24 hours after treatment (Fig. 3c) and there was a progressive increase in the number of foci per cell over this dose range (Fig. 3d-f). Representative images of MNNG-treated cells show the heterogeneity in foci number per nucleus (Fig. 3g-i). Therefore unlike the neutral comet assay, retention of γH2AX foci appears to have the requisite sensitivity to predict cell survival over the first log or two of cell killing.

### Ability of residual γH2AX to predict clonogenic survival after exposure to other drugs

**Figure 4:**
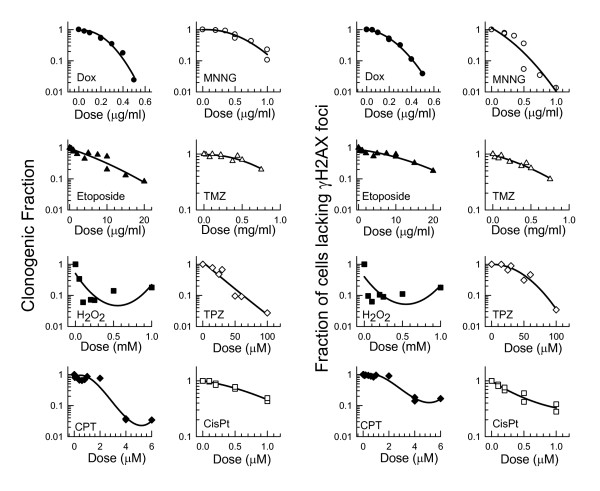
**Comparison between clonogenic fraction and fraction of cells lacking residual γH2AX foci for SiHa cells exposed to 8 drugs**. With the exception of tirapazamine treatment that was conducted in suspension culture under anoxia, attached cells were exposed at 37°C in complete medium for either 30 min or 60 min (cisplatin, temozolomide). After rinsing, cells were allowed to recover for 24 hours before microscopic analysis of γH2AX foci and plating to measure clonogenic fraction. Combined results from 2-4 experiments are shown.

To determine whether the fraction of cells with residual γH2AX foci would predict surviving fraction for other drugs, the response of SiHa cells to a broader range of drugs was subsequently examined. Drug concentrations that typically produced no more than 1 log of cell kill were used to avoid rapid cell loss often associated with higher drug doses [22]. In all cases, SiHa cells were exposed for 30 or 60 min to each drug and incubated for 24 hours after drug treatment to allow time for DNA repair and concomitant loss of γH2AX foci. After 24 hours, cells were plated for measurement of clonogenic fraction or fixed for measurement of the fraction of cells lacking residual γH2AX foci. Clonogenic fraction and the relative fraction of cells lacking foci 24 h after exposure showed similar dose response patterns for each drug (Fig. 4). The slopes comparing clonogenicity with residual foci together with correlation coefficients are provided in Table 1. The 95% confidence limits on the slopes included the value of one for 7/8 drugs, indicating that the fraction of cells that retained residual γH2AX foci was directly comparable to the fraction of cells that would die. The lowest surviving fraction produced by temozolomide was only 0.58 which may have been insufficient to accurately estimate the slope. These results indicate that residual γH2AX is useful for predicting clonogenic fraction following exposure to a broad range of genotoxic drugs. This suggests, but does not prove, that cells that retain γH2AX foci 24 hours after drug treatment are likely to die.

**Table 1:** Correlation between residual γH2AX and cell killing in SiHa monolayers exposed to DNA damaging agents

DNA damaging agent	Mechanism of interaction with DNA	Likely mechanism(s) of γH2AX formation	^a^Slope (95% confidence limits) and correlation coefficient (r^2^)
**X-rays**	SSB, DSB, base damage	Direct DSBs	**0.95 (0.57-1.33)** **r^2 ^= 0.95**

**Etoposide**	Topo II inhibitor	Direct DSBs	**1.17 (0.91-1.44)** **r^2 ^= 0.91**

**Temozolamide**	Base alkylation	Base damage leading to DSBs during replication^b^	**0.62 (0.42-0.82)** **r^2 ^= 0.82**

**MNNG**	Base alkylation	Base damage leading to DSBs during replication^b^	**0.79 (0.52-1.07)** **r^2 ^= 0.87**

**Cisplatin**	Inter- and intra-strand crosslinks	Inter- and intra-strand crosslinks leading to DSBs during replication^b^	**0.77 (0.53-1.02)** **r^2 ^= 0.87**

**Doxorubicin**	SSB, DSB, base damage, topo II inhibitor	Direct DSBs and replication fork blockage leading to DSBs	**0.95 (0.88-1.03)** **r^2 ^= 0.99**

**Tirapazamine**	SSB, DSB, base damage, topo II inhibitor	Direct DSBs and replication fork blockage leading to DSBs	**1.06 (0.80-1.3)** **r^2 ^= 0.90**

**Camptothecin**	Topo I inhibitor	Replication fork blockage leading to DSBs	**0.83 (0.61-1.04)** **r^2 ^= 0.85**

**Hydrogen peroxide**	SSB	Opposed single-strand breaks	**1.06 (0.85-1.26)** **r^2 ^= 0.93**

^a ^The slope was obtained by plotting clonogenic surviving fraction versus the fraction of cells lacking foci.

^b ^After exposure to very high drug doses, γH2AX forms independently of cell cycle phase and may implicate opposed repair sites as responsible for triggering H2AX phosphorylation.

### Residual RAD51 foci predict cell response to radiation

**Figure 5:**
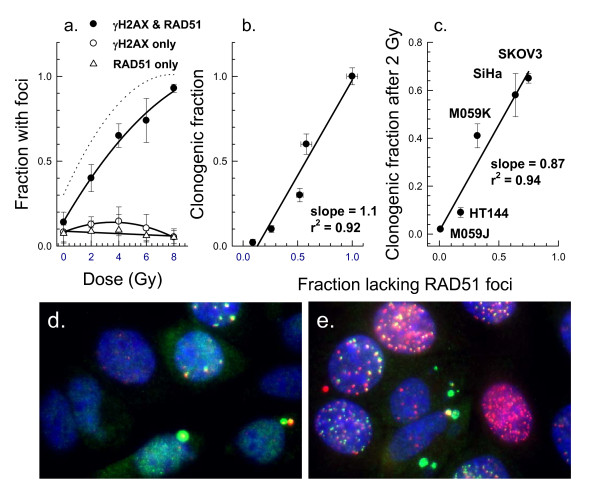
**The fraction of cells with residual RAD51 foci is correlated with the fraction of cells that die**. Panel a: SiHa cells were exposed to X-rays and allowed to recover for 24 hours. Cells were fixed and co-immunostained for γH2AX and RAD51, and cells with foci were scored. Results are the means and SD for 3 experiments. Panel b: The fraction of SiHa cells lacking RAD51 foci is compared with the clonogenic fraction measured 24 hours after exposure to X-rays. Panel c: Several cell lines were exposed to 2 Gy, allowed to recover for 24 hours, and then examined for the fraction of cells that lacked RAD51 foci. Panel d: RAD51 (green) and γH2AX (red) antibody staining of SiHa cells 24 hours after exposure to 2 Gy. Nuclei are stained blue with DAPI. Panel e: 24 hours after exposure to 8 Gy. Note the micronuclei stained with antibodies to RAD51 and/or γH2AX foci and the co-localization of some foci in some cells but not all cells.

As it was not possible to confirm directly that cells that retained γH2AX foci 24 hours after treatment were the cells that died, we attempted to accomplish this in another way by using live-cell analysis of SiHa cells expressing RAD51-GFP. First, it was important to confirm results of a previous study that showed that the fraction of cells that died after irradiation was correlated with the fraction of cells with RAD51 foci 24 hours after irradiation [6]. Exponentially growing SiHa cells were exposed to ionizing radiation and allowed to recover for 24 hours before plating for survival or fixing in 2% paraformaldehyde for analysis of RAD51 and γH2AX foci. The fraction of SiHa cells that expressed γH2AX and RAD51 foci 24 hours after irradiation increased with dose, and the majority of foci-positive cells exhibited both foci (Fig. 5a), although not necessarily co-localized. About 5-10% of the cells that lacked RAD51 foci exhibited γH2AX foci, but <5% of cells showed RAD51 foci in the absence of γH2AX foci. As mitotic cells did not exhibit RAD51 foci, this could account in part for the presence of residual γH2AX foci in the absence of RAD51 foci. Differences in rate of foci development and removal may also account for minor discrepancies. The correlation between the fraction of SiHa cells that lacked RAD51 foci and surviving fraction after irradiation was excellent (Fig. 5b). To determine whether this correlation would hold for other cell types, several cell lines were exposed to 2 Gy and examined for the presence of RAD51 foci 24 hours after irradiation. In agreement with results of Sak et al. [6], the fraction of cells that exhibited RAD51 foci 24 hours after exposure to 2 Gy was higher for the more radiosensitive cell lines (Fig. 5c). RAD51 foci appeared in many micronuclei 24 hours after exposure, either alone or with γH2AX foci, and daughter cell pairs showed similar RAD51 foci patterns as has been reported for γH2AX foci (Fig 5d, e). Although RAD51 foci may only mark a subset of the double-strand breaks, the number of residual RAD51 foci per cell was not invariably lower than the number of residual γH2AX foci (Fig. 5e). Therefore, residual RAD51 foci appear to behave much like residual γH2AX foci, at least at 24 hours post-treatment, and should therefore be useful for predicting response to treatment.

### Use of RAD51-GFP transfected cells to determine cell fateafter irradiation

**Figure 6:**
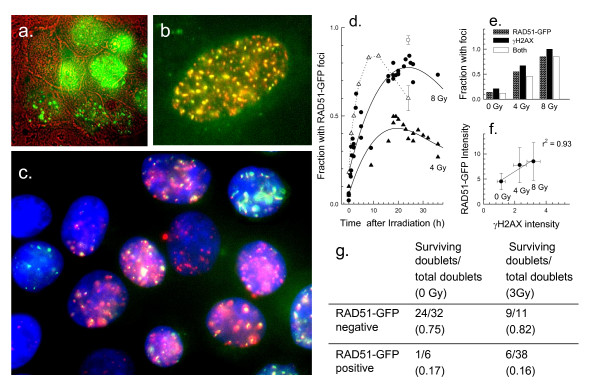
**RAD51-GFP as a live cell marker**. Panel a: RAD51-GFP foci in live SiHa cells stably transfected with RAD51-GFP are shown 24 hours after exposure to 8 Gy. The phase contrast image is indicated by red outlines. Panel b: Co-localization between RAD51-GFP and anti-RAD51 antibody staining (red) in an untreated SiHa cell. Panel c: Co-localization between RAD51-GFP (green) and γH2AX antibody staining (red) in SiHa cells 24 hours after exposure to 8 Gy. Panel d: Analysis of the fraction of SiHa-RAD51-GFP cells that develop foci as a function of time after exposure to 4 Gy or 8 Gy. Results from several experiments are pooled. The dotted line and open circles show the development of RAD51 antibody-labelled foci after exposure to 4 Gy. The single open triangle shows the fraction of RAD51 antibody-labeled cells 24 hours after 8 Gy. Panel e: The fraction of cells that exhibit RAD51-GFP foci or γH2AX foci 24 hours after exposure to radiation, measured microscopically. Panel f: SiHa-RAD51-GFP cells expressing high levels of RAD51-GFP after 24 hours after irradiation were sorted on the basis of GFP, fixed and stained for γH2AX. The average intensity of the populations was measured using flow cytometry. The mean and standard error for 3 sorted populations is shown. Panel g: Clonogenicity of SiHa-RAD51-GFP cells after 0 or 3 Gy exposure. Cells in 8-well dishes were irradiated and 24 hours later, wells containing one or two doublets were scored for the presence or absence of γH2AX foci (daughter cell pairs show the same foci patterns). Dishes were returned to the incubator for 2 weeks to form colonies. The fraction of doublets with foci that survived treatment or the fraction lacking foci that survived treatment was calculated.

Having established that the fraction of cells that retain RAD51 foci is similar to the fraction of cells that die after irradiation, SiHa cells were stably transfected with a plasmid containing a RAD51-GFP reporter construct. A clone with moderate RAD51-GFP expression (GFP expression was 3.9 times background) that showed similar growth kinetics and radiation response as the parental cell line was selected (data not shown). Detection of individual GFP-labelled foci was possible using live cells (Fig. 6a), and excellent co-localization was seen between RAD51-GFP and RAD51 antibody staining (Fig 6b). Although RAD51-GFP filament formation was occasionally observed, the majority of cells with RAD51-GFP foci showed a punctuate pattern, and cells with RAD51-GFP foci typically expressed γH2AX foci (Fig. 6c). The number of cells with RAD51-GFP foci reached a maximum 16 - 24 h after irradiation (Fig. 6d), exhibiting kinetics similar to those reported using immunoblotting [23] but slower than observed using immunofluorescence staining (dotted line in Fig. 6d). This difference is likely to be a resolution issue since sufficient RAD51-GFP molecules must aggregate to become microscopically visible. The fraction of cells with microscopically visible RAD51-GFP foci was similar to the fraction of cells with antibody-labeled RAD51 foci 24 hours after 4 or 8 Gy (Fig. 6d). In SiHa cells exposed to 4 or 8 Gy, the fraction of cells with RAD51-GFP foci was also similar to the fraction of cells with γH2AX foci when detected microscopically (Fig. 6e). In SiHa cells sorted on the basis of RAD51-GFP 24 h after irradiation and then stained and reanalyzed for γH2AX using flow cytometry, there was a good correlation between the expression of these two molecules (Fig. 6f). Therefore RAD51-GFP expression appears to be a useful surrogate for γH2AX expression when analyzed 24 hours after treatment.

To determine whether SiHa cells that exhibit RAD51-GFP foci 24 hours after irradiation are more likely to die, SiHa-RAD51-GFP cells were exposed to 0 or 3 Gy. Twenty-four hours later, daughter-cell doublets were scored for the presence or absence of GFP foci. Preliminary results established that 58% of unirradiated cells formed doublets by 24 hours and a similar percentage (50%) of those exposed to 3 Gy formed doublets. For control cells, 95% of the doublets lacked foci at 24 hours and for the irradiated cells, 60% lacked foci. After allowing 2 weeks for doublets to form colonies, wells were scored. Only 16% of the doublets with RAD51-GFP foci after 24 h formed colonies whether they were exposed to 0 or 3 Gy (Fig. 6g) whereas 75-82% of doublets without foci were able to form colonies. These results support the hypothesis that cells that retain foci 24 hours after irradiation are the cells that lose clonogenic potential.

## Discussion

We have previously reported that the fraction of SiHa cells that exhibit more than the background level of γH2AX 24 hours after treatment can be correlated with the fraction of cells that will ultimately die after exposure to X-rays and/or cisplatin [5,24]. We now show that retention of γH2AX foci can predict clonogenicity after exposure to a variety of DNA damaging drugs, several of which do not produce direct DSBs. DSBs can be formed indirectly by two closely opposing single-strand breaks. This is likely to be the case with hydrogen peroxide since sufficiently high drug doses will produce DSBs [25]. MNNG-induced double-strand breaks were also detected using the neutral comet assay after exposure to high doses (Fig. 2e), and closely opposed damaged sites, perhaps undergoing base excision repair could be responsible [26]. However, as a high lysis temperature was used for the neutral comet experiments, opposing heat-labile base damage sites may also be involved in the formation of physical breaks detected using this method [27].

Several patterns of γH2AX formation and loss have been observed using the drugs listed in Table 1. Foci reach a maximum size within an hour after exposure to X-rays, tirapazamine, doxorubicin or etoposide. However, development of γH2AX is slower when DSB formation requires transit through S phase, for example, after treatment with cisplatin or low doses of MNNG. Significant DSB rejoining was not detected during the 24 hours after treatment with MNNG and γH2AX levels also remained high (Fig. 2c). The inability to repair DSBs caused by MNNG was also suggested by Stojic et al, [28] based on the persistence of γH2AX foci in MNNG treated cells. Unfortunately, the presence of an unrejoined double-strand break at the site of a residual γH2AX focus cannot be confirmed using a physical method to detect DSBs since these methods lack sufficient sensitivity. A subsequent paper by Stojic et al. indicated that a different mechanism operated to induce γH2AX foci after exposure to high MNNG doses (30 μM) because foci formation became independent of mismatch repair which was associated with replication [29]. Our flow cytometry results (Fig. 1) support this observation by indicating that cell cycle position is important for γH2AX focus formation after exposure to low but not high (> 20 μg/ml) MNNG doses.

Although our results suggest that residual, not initial γH2AX is the critical factor determining cell fate, initial γH2AX can also be predictive for response if some cells within a population are resistant to the induction of DBSs. Etoposide causes DSBs only in the outer proliferating cells of multicellular spheroids; the non-proliferating inner fraction of cells do not develop γH2AX and therefore survive treatment [30]. In this case, determining the fraction of cells lacking γH2AX immediately after exposure was also predictive of cell survival [31]. In the same way, doxorubicin penetrates poorly through the outer cells of spheroids so that only the outer cells developed significant numbers of γH2AX foci. Again the fraction of cells lacking foci immediately after exposure was correlated with the fraction of cells that survived [31]. However, by counting the fraction of cells lacking foci 24 hours after treatment, the importance of repair capacity as well as susceptibility to a direct-acting genotoxin can be included in the estimate of survival. Moreover, effects of drugs that produce γH2AX only when cells transit S phase can also be evaluated, provided of course that drug-treated cells are given an opportunity to progress through the cell cycle.

A RAD51-GFP construct provided a way to directly address the importance of residual DNA repair foci in determining cell fate. Cells deficient in H2AX also show a deficiency in homologous recombination and RAD51 focus formation [32,33], and it is possible that retention of γH2AX may be the signal for retention of repair molecules like RAD51 [10]. Most but not all cells with RAD51-GFP foci 24 hours after irradiation failed to form colonies. Similarly, most but not all cells lacking foci did form colonies (Fig. 6g). Unfortunately, resolution for detecting foci was reduced under the technical constraints imposed by live cell imaging in multiwells, and 24 hours may not have been an optimum time to score microscopically visible RAD51-GFP foci for all cells. Although the answer was not unequivocal, it does support the idea that residual DNA repair foci mark cells that are likely to die. Since all of the DNA damaging agents we have examined produced residual γH2AX that were predictive of clonogenic survival, it should be possible to use residual foci as a biomarker of response to genotoxic agents.

There are limitations to the application of this approach *in vivo*. Tumor heterogeneity is a major consideration especially since both induction of DNA damage and its repair are influenced by the tumor microenvironment. Obtaining a representative biopsy and/or multiple biopsies will be essential [34]. Second, it is important to consider the endogenous expression of γH2AX since this can be quite variable and will affect the ability to detect residual γH2AX foci [35]. A pre-treatment biopsy must also be obtained and if endogenous γH2AX is excessive, prediction based on residual foci may not be possible. Third, loss of heavily damaged cells by apoptosis or other mechanisms within the first 24 hours after treatment, or sequestration of foci into micronuclei before scoring will reduce the accuracy of prediction. Although early apoptotic cells exhibit γH2AX foci, necrosis secondary to apoptosis can result in loss of the signal [16,36]. Finally, for drugs that produce foci only when DNA replicates, it will be necessary to ensure that all treated cells have the opportunity to transit S phase. In spite of these limitations, the recent application of γH2AX to predict response to cisplatin combined with radiation in xenograft tumors [24] indicates that this approach has promise for early prediction of tumor response to treatment. Moreover, it should be possible to predict response not only to single drugs but to combinations of DNA damaging agents.

## Conclusions

Our results support the hypothesis that tumor cells that retain γH2AX foci 24 hours after exposure to a DNA damaging agent are unlikely to survive treatment. The direct relationship between loss of clonogenic ability and retention of γH2AX foci holds for drugs that damage DNA by different mechanisms. Therefore, it should be possible to identify drug-resistant tumor cells simply by measuring the fraction of cells that lack residual γH2AX foci.

## Competing interests

The authors declare that they have no competing interests.

## Authors' contributions

JPB performed comet assays and the comparisons between γH2AX foci and survival. SHM created the RAD51-GFP transfected cell line which was characterized by DK and CAB. PLO conceived the study and wrote the paper. All authors contributed to study design and data analysis and approved the final manuscript.

## Pre-publication history

The pre-publication history for this paper can be accessed here:

http://www.biomedcentral.com/1471-2407/10/4/prepub
